# Study on the Image Recognition of Field-Trapped Adult *Spodoptera frugiperda* Using Sex Pheromone Lures

**DOI:** 10.3390/insects16090952

**Published:** 2025-09-11

**Authors:** Quanyuan Xu, Caiyi Li, Min Fan, Ying Lu, Hui Ye, Yonghe Li

**Affiliations:** 1 College of Big Data and Intelligent Engineering, Southwest Forestry University, Kunming 650224, China; xuqy@swfu.edu.cn (Q.X.);; 2 Key Laboratory of Forestry and Ecological Big Data State Forestry Administration, Southwest Forestry University, Kunming 650024, China; 3 Huize County Bureau of Agriculture and Rural Affairs, Qujing 654200, China; 4 School of Ecology and Environment, Yunnan University, Kunming 650091, China; 5 Yunnan Plateau Characteristic Agricultural Industry Research Institute, Yunnan Agricultural University, Kunming 650210, China

**Keywords:** *Spodoptera frugiperda*, Mask R-CNN, YOLOv5, trapping posture adults, image recognition

## Abstract

*Spodoptera frugiperda* is a major transboundary migratory pest under global alert by the Food and Agriculture Organization (FAO) of the United Nations. The accurate identification and counting of trapped adults in the field are key technologies for achieving quantitative monitoring and precision pest control. However, precise recognition is challenged by issues such as scale loss and the presence of mixed insect species in trapping images. To address this, we constructed a field image dataset of trapped *Spodoptera frugiperda* adults and proposed an improved YOLOv5s-based detection method. The dataset was collected over a two-year sex pheromone monitoring campaign in eastern–central Yunnan, China, comprising 9550 labeled insects across six categories, and was split into training, validation, and test sets in an 8:1:1 ratio. In this study, YOLOv7, YOLOv8, Mask R-CNN, and DETR were selected as comparative baselines to evaluate the recognition of images containing *Spodoptera frugiperda* adults and other insect species. However, the complex backgrounds introduced by field trap photography adversely affected classification performance, resulting in a relatively modest average accuracy. Considering the additional requirement for model lightweighting, we further enhanced the YOLOv5s architecture by integrating Mosaic data augmentation and an adaptive anchor box strategy. Additionally, three attention mechanisms—SENet, CBAM, and Coordinate Attention (CA)—were embedded into the backbone to build a multidimensional attention comparison framework, demonstrating CBAM’s superiority under complex backgrounds. Ultimately, the CBAM-YOLOv5 model achieved 97.8% mAP@0.5 for *Spodoptera frugiperda* identification, with recognition accuracy for other insect species no less than 72.4%. Based on the optimized model, we developed an intelligent recognition system capable of image acquisition, identification, and counting, offering a high-precision algorithmic solution for smart trapping devices.

## Simple Summary

*Spodoptera frugiperda* is a globally significant migratory pest. The accurate identification and counting of trapped adults is essential for precise monitoring and control. This study constructs a two-year field image dataset in Yunnan, China, and proposes an improved YOLOv5s-based detection model. In comparison with YOLOv7, YOLOv8, Mask R-CNN, and DETR, which exhibit relatively lower average accuracies and considerably larger parameter scales, the optimized YOLOv5s—integrating Mosaic augmentation, adaptive anchor boxes, and attention mechanisms—achieves high average accuracy while maintaining a compact model size. Among SENet, CBAM, and CA, CBAM performed best under complex conditions. The CBAM-YOLOv5 model achieved 97.8% mAP@0.5 with no false positives and over 72.4% accuracy for other insects. An intelligent system was developed for image capture, recognition, and counting. This work supports smart pest monitoring and contributes to sustainable agricultural practices.

## 1. Introduction

*Spodoptera frugiperda*, belonging to the order *Lepidoptera*, family *Noctuidae*, and genus *Spodoptera* [1], is a transboundary migratory agricultural pest listed on the global alert system of the Food and Agriculture Organization (FAO) of the United Nations. Native to tropical regions of the Americas, it has now spread to over 100 countries across Africa, Asia, and beyond [2]. Adult *S. frugiperda* are capable of long-distance migration, often covering several hundred kilometers in a single flight, making them a primary vector for regional pest outbreaks. Under optimal conditions (25 °C), a single female moth can lay between 800 and 1800 eggs, with an average of 1482, and has an egg hatching rate exceeding 95%. This leads to exponential population growth during the larval stage, characterized by voracious feeding behavior that can result in 20–70% crop yield losses. Staple crops such as maize and rice are particularly vulnerable to such destructive infestations [3]. Monitoring adult *S. frugiperda* populations using pheromone trapping plays a vital role in forecasting population dynamics, disrupting reproductive chains, and implementing integrated pest management strategies. In particular, the accurate identification and quantification of trapped adults serve as critical technical foundations for quantitative surveillance and precision control of this invasive pest.

However, field-based monitoring of *S. frugiperda* adults faces three critical technical bottlenecks. First, due to the morphological similarity between *S. frugiperda* and other pests (e.g., Mythimna separata), field surveys reveal that many farmers misidentify the species, leading to the misuse of pesticides and heightened ecological risks [4]. Second, mainstream monitoring methods suffer from inherent limitations: pheromone traps exhibit high false detection rates due to interference from non-target insects, while both pheromone- and light-based monitoring techniques offer insufficient classification accuracy and low efficiency in field deployments [5,6]. Third, current AI-based recognition models heavily rely on standardized laboratory samples, whereas in field conditions, the scales of trapped adults detach progressively with prolonged struggle. The rate of scale loss increases linearly with trapping duration, and, after 72 h, wing surface patterns become severely degraded, resulting in a significant decline in recognition accuracy [7].

Field monitoring of *S. frugiperda* using pheromone traps presents multiple challenges. Firstly, the trapped adults often exhibit highly variable morphological features due to scale loss, and are frequently intermixed with a variety of non-target insect species. Secondly, the process of capturing images of trapped specimens within the collection buckets is subject to numerous uncontrolled variables. These include inconsistent camera models, leading to significant differences in image quality; uneven image resolution; uncontrollable lighting conditions; varying shooting distances (i.e., from the lens to the bottom of the bucket); diverse shooting angles; and complex, cluttered backgrounds. As a result, the collected images differ substantially in clarity and overall quality, complicating subsequent recognition tasks.

Traditional insect recognition methods have commonly employed classical machine learning algorithms such as Support Vector Machine (SVM) [8], Decision Tree (DT) [9], and Random Forest (RF) [10], in combination with handcrafted feature extraction techniques including the Gray-Level Co-occurrence Matrix (GLCM) [11], Histogram of Oriented Gradient (HOG) [12], and Local Binary Pattern (LBP) [13]. For instance, Larios et al. [14] utilized Haar features in conjunction with an SVM classifier to achieve high-accuracy identification of stonefly larvae. Zhu Leqing et al. [15] extracted OpponentSIFT descriptors and color information features from *Lepidoptera* specimens and successfully classified them using SVM. Liu Deying et al. [16] performed binary processing on dorsal images of rice planthoppers and applied logical AND operations with grayscale images to extract discriminative parameters, achieving a recognition accuracy exceeding 90%. Xiao et al. [17] combined Scale-Invariant Feature Transform (SIFT) descriptors with a Bag-of-Features framework and classified vegetable pest images using SVM, reaching an accuracy of 91.6% with an inference speed of 0.39 s per image, thereby demonstrating suitability for real-time applications in precision agriculture. Bakkay et al. [18] proposed a multi-scale feature extraction approach based on HCS descriptors, coupled with SVM, to detect and count moths in trap images, achieving an accuracy of 95.8%.

In 2006, Hinton et al. [19] introduced an unsupervised greedy layer-wise training algorithm for Deep Belief Networks (DBNs) [20], which addressed key challenges in improving deep learning accuracy and significantly accelerated the advancement of the field. Watson et al. [21] developed an automated identification system for 35 species of *Lepidoptera* using Convolutional Neural Networks (CNNs), achieving an identification accuracy of 83% under natural field conditions. Proença et al. [22] applied YOLOv5 to identify green leafhoppers trapped on yellow sticky boards in vineyards, attaining a precision of 99.62% with an average inference time of approximately 2.5 s per image, thereby reducing manual labor costs. Zhang Yinsong et al. [23] enhanced the Faster R-CNN framework by replacing VGG16 with ResNet50 and employing Soft-NMS in place of traditional non-maximum suppression, achieving a detection accuracy of 90.7%. Li et al. [24] combined CNNs with non-maximum suppression techniques to locate and count rice aphids, obtaining 93% accuracy with an mAP of 0.885. Malathi et al. [25] proposed a tri-channel T-shaped deep CNN (T-CNN) for rapid identification of *S. frugiperda*, establishing an effective recognition technique. Feng et al. [26] developed a CNN-based detection method for *S. frugiperda* on maize leaves, validating the use of a separable attention mechanism within ResNet50 to enhance both accuracy and robustness. Zhang et al. [27] introduced a two-stage classification network, MaizePestNet, integrating Grad-CAM and knowledge distillation strategies to identify 36 species of adult and larval maize field pests, achieving a precision of 93.85%. Although recent studies have achieved notable improvements in the recognition accuracy of *S. frugiperda*, research on its monitoring remains limited. Despite the widespread use of pheromone-based trapping, there is a lack of dedicated studies on image-based identification from trap captures. Furthermore, the loss of wing scales following entrapment often degrades key morphological features, thereby hindering effective feature extraction and reducing recognition accuracy. To address these challenges, it is imperative to construct a field-based, high-quality dataset that reflects real monitoring conditions in order to enhance the practical applicability of deep learning models for pest surveillance.

This study departs from conventional research paradigms by constructing a dynamic field-based image dataset of pheromone-trapped insects, comprising 9550 annotated specimens, and proposing an enhanced YOLOv5 detection framework. The key innovations of this work are threefold: (1) A multi-stage data augmentation strategy is designed to specifically address the degradation of morphological features caused by scale loss in trapped moths; (2) a channel–spatial attention coordination mechanism (CBAM) is introduced to suppress background interference while compensating for degraded insect features; and (3) a lightweight insect counting algorithm is developed, demonstrating strong real-time processing capabilities.

Comprehensive comparative experiments confirm that the improved model achieves a mAP@0.5 of 97.8% even under trapping conditions involving 72-h scale loss. These results provide a highly accurate and deployable algorithmic solution for intelligent pest-trapping devices.

## 2. Materials and Methods

### 2.1. Data Acquisition and Preprocessing

#### 2.1.1. Data Source and Acquisition

In this study, adult *S. frugiperda* specimens captured using sex pheromone traps were selected as the target objects for image-based detection. A multi-source dataset was constructed, comprising three categories: (1) field images of pheromone traps photographed manually, serving as the core training samples; (2) images of *S. frugiperda* adults reared under standardized laboratory conditions; and (3) publicly available adult moth images obtained from online biodiversity repositories. This combination ensured a broadly representative and diverse sample collection.

The field images were acquired from pheromone traps deployed in maize fields across eastern and central Yunnan Province, China (101°16′–106°12′ E, 22°26′–27°03′ N). A total of 96 pheromone monitoring sites were established between 2021 and 2022. The traps consisted of PVC-based pheromone lures placed in bucket-shaped traps, installed in maize cultivation areas. Monitoring personnel—including local farmers and agricultural technicians, used smartphones to photograph the bottom of the traps once or twice per week, capturing digital images in JPG format. After each photographic session, the trapped insects were removed from the container to maintain trap hygiene and prevent duplication of specimens.

To enhance data diversity, two additional image sources were incorporated:

① *S. frugiperda* adults reared in controlled laboratory environments at the Institute of Biodiversity Conservation, Southwest Forestry University, Kunming, China, photographed under standardized conditions using smartphones by trained researchers;

② standardized specimen images obtained from international biodiversity platforms. The data from the Global Biodiversity Information Facility (GBIF) were accessed via a specific occurrence download [28]. Additional images were sourced from the Centre for Agriculture and Bioscience International (CABI).

**Figure 1:**
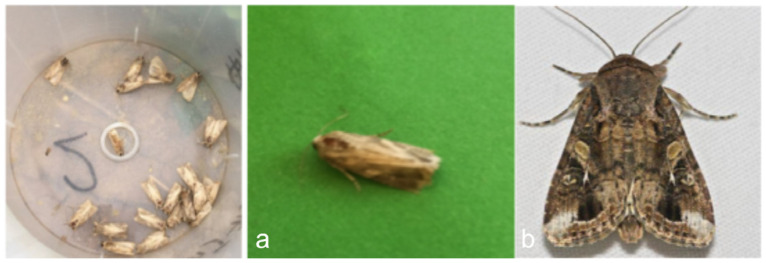
Lab shooting and web access. (**a**) Pictures taken in the laboratory. (**b**) Pictures obtained from the Internet.

Representative samples from the pheromone-trap images, laboratory images, and web-sourced specimens are shown in Figure 1.

#### 2.1.2. Data Preprocessing and Allocation

During field-based pheromone trapping of *S. frugiperda* adults, the moths captured in the collection buckets frequently exhibit complex and variable patterns of scale loss. In addition, a diverse mixture of insect species is commonly present within the trap. The use of sex pheromones enables the selective attraction of *S. frugiperda*, but the adult moths often struggle inside the trap, leading to a time-dependent linear increase in scale loss. After 72 h of confinement, approximately 58% of wing surface texture features are lost due to scale detachment.

The degree of scale degradation in trapped moths can be classified into three categories: mild, moderate, and complete loss. Moreover, additional morphological challenges frequently occur, such as occlusion and overlap between individuals, as well as body inversion following death, further complicating accurate recognition. Representative examples of these phenomena are summarized in Table 1.

In addition to *S. frugiperda*, sex pheromone traps in the field often capture a variety of non-target insect species. These include commonly encountered pests such as *Mythimna loreyi*, *Spodoptera litura*, *Apis* spp., and *Melolonthidae sensu stricto*. Other insects captured in smaller quantities include lady beetles *Coccinellidae*, *Acridoidea*, and *Papilionoidea*, among others.

The process of photographing trapped insects within the buckets introduces multiple uncontrolled variables. These include inconsistent imaging quality due to the use of different smartphone models by monitoring personnel, varying image resolutions, uncontrollable lighting conditions, inconsistent shooting distances (relative to the bottom of the trap), diverse viewing angles, and complex backgrounds. As a result, the acquired images differ markedly in clarity and overall quality.

**Figure 2:**
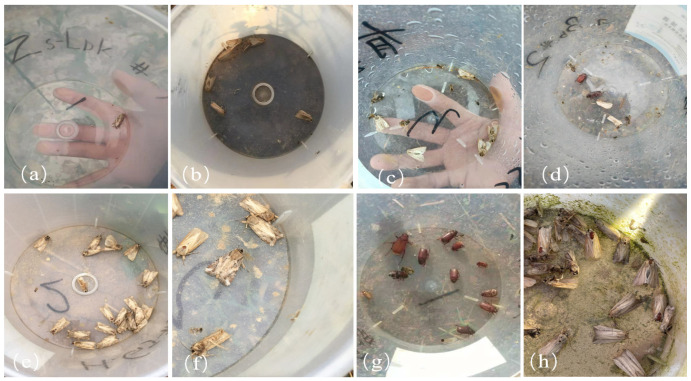
Trap image. (**a**) Single-target trapping image. (**b**) Multiobjective *Mythimna loreyi*. (**c**) Multi-object multi-class images. (**d**) Multiple categories and multiple objectives. (**e**) A single category with multiple objectives. (**f**) A single class with multiple targets, with flaking. (**g**) Multiple classes and multiple targets, with different target insect sizes. (**h**) Multi-object multi-category, complex background, and mutual occlusion.

Examples of field-collected images obtained from pheromone traps are shown in Figure 2. These images may contain single or multiple insects, either of a single species or across multiple species. Moreover, the moths often exhibit varying degrees of scale loss, diverse postures, and complex visual backgrounds. These factors substantially increase the difficulty of accurate recognition, posing considerable challenges to improving detection precision for trapped *S. frugiperda* adults.

To address the issues of image quality defects and class imbalance inherent in the raw dataset, this study conducted a systematic preprocessing of the multi-source *S. frugiperda* image collection. Given the irregular spatial distribution of target insects within the trap images, precise target region cropping was performed through manual annotation, effectively reducing background interference and significantly enhancing the discriminability of target features. Data analysis revealed a pronounced class imbalance in the original dataset: *S. frugiperda* samples accounted for 67% (4673 images), approximately 2.03 times the combined total of the other five insect categories. Such skewness severely hampers the classification performance of machine learning models.

To mitigate this issue, a novel multi-strategy data augmentation approach was proposed. Minority class samples were augmented using a randomized combination of eleven operations, including geometric transformations (flipping, translation), color space adjustments (gamma correction, brightness modulation), and spatial filtering techniques (Gaussian smoothing, edge enhancement), effectively enriching data diversity. After augmentation, the dataset size was expanded to 9550 images, with a more balanced class distribution as summarized in Table 2. The final curated dataset comprises 2769 high-quality images, which were partitioned into training (2215 images), validation (277 images), and test (277 images) sets at an 8:1:1 ratio, providing a robust foundation for subsequent model training.

#### 2.1.3. Statistical Analysis of the Number of Images in the Dataset

**Figure 3:**
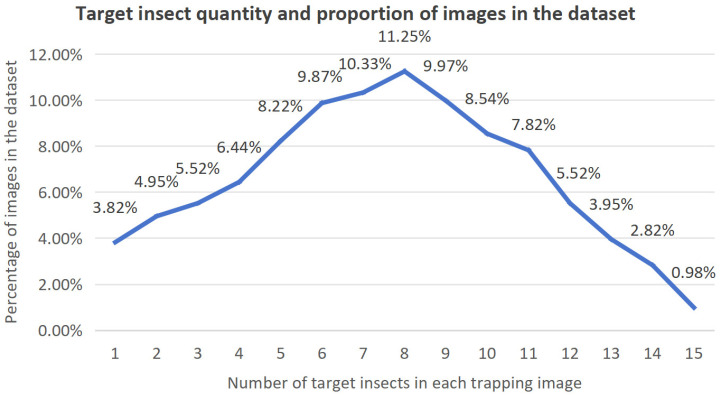
The number of target insects in each trapping image and the proportion of the number of images in the dataset.

To enhance the quality of the dataset in terms of both data diversity and category richness, we collected image data under varying photographic environments, carefully accounting for the potential influence of background complexity and illumination conditions. Specifically, images were captured across four distinct environmental settings, with the proportional distribution of samples from each environment summarized in Table 3. Furthermore, recognizing the challenge posed by the uneven spatial distribution of target insects within images, we deliberately incorporated scenarios involving single-class versus multi-class samples, as well as single-target versus multi-target instances. Consequently, a comprehensive insect dataset was established, in which each trap image contains between one and fifteen targets. The proportion of images corresponding to different target quantities within the dataset is illustrated in Figure 3.

#### 2.1.4. Data Annotation Processing

**Figure 4:**
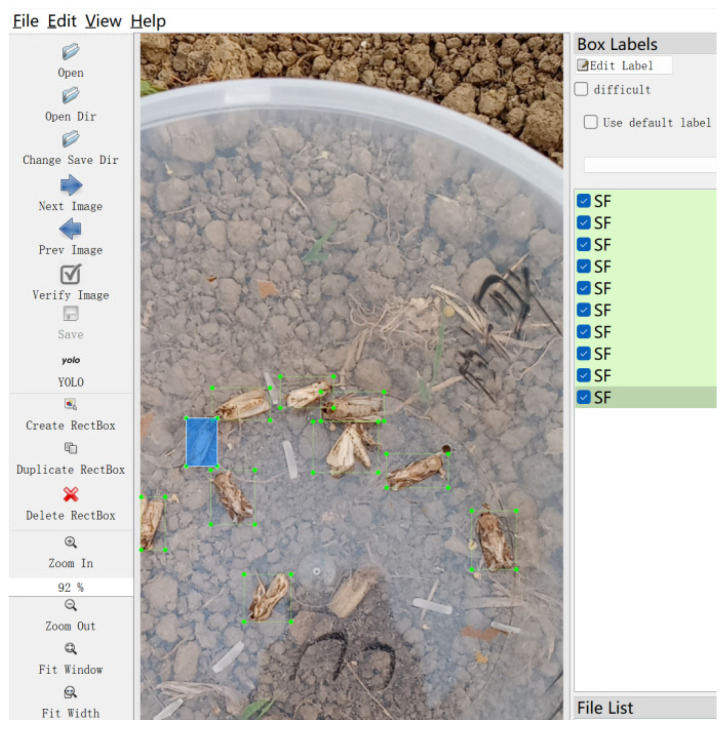
Data annotation tool (In the figure, the blue area represents the range of regions used to select targets when annotating data).

**Figure 5:**
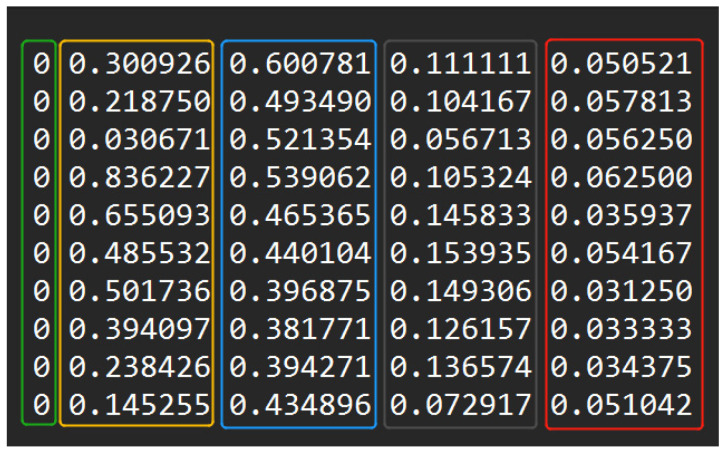
Annotate file storage information (Green represents the category ID; yellow represents the normalized x-coordinate of the bounding box center relative to the image width; Blue represents the normalized y-coordinate of the bounding box center relative to the image width; Gray corresponds to the normalized bounding box width relative to the image width; Red represents the normalized bounding box height relative to the image height.).

In this study, the LabelImg (v1.8.1) software was employed for manual image annotation. Six categories were defined, namely SF (*Spodoptera frugiperda*), ZC (*Mythimna loreyi*), XY (*Spodoptera litura*), MF (*Apis*), JC (*Melolonthidae sensu stricto*), and Other (miscellaneous insect species). An example of the annotated images is presented in Figure 4. The folders containing the target images were imported into the annotation software, with the labeling mode specified as YOLO format. Rectangular bounding boxes were drawn around the insects of interest to encode both their positional information and category labels, after which a corresponding .txt annotation file was generated. Each text file recorded the annotations for a single image, with one line representing one annotated object, as illustrated in Figure 5. Each annotation entry consists of five components: the green-marked section denotes the category ID; the yellow section represents the normalized x-coordinate of the bounding box center relative to the image width; the blue section represents the normalized y-coordinate of the center relative to the image height; the gray section corresponds to the normalized bounding box width relative to the image width; and the red section denotes the normalized bounding box height relative to the image height.

### 2.2. Building the Model

#### 2.2.1. YOLOv5

**Figure 6:**
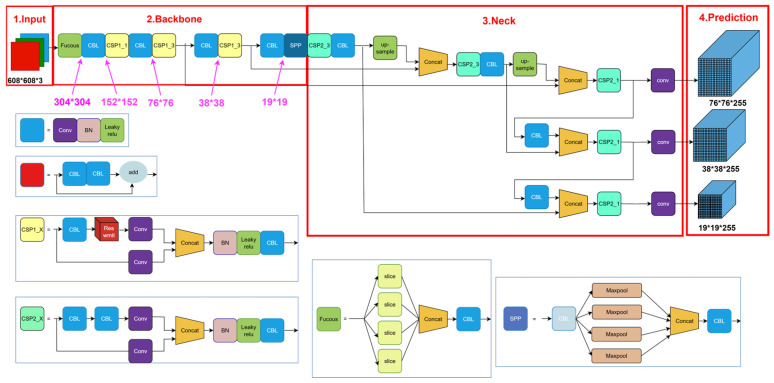
YOLOv5 network model structure.

YOLOv5 is a deep learning algorithm designed for object detection, available in four variants: YOLOv5s, YOLOv5m, YOLOv5l, and YOLOv5x. In this study, YOLOv5l was selected as the baseline model. As illustrated in Figure 6, the architecture of YOLOv5 consists of four main components: the input layer, the backbone, the neck, and the prediction head.

Input Stage: YOLOv5 accepts input images of arbitrary dimensions, provided that both the height and width are multiples of 32. In this study, the input image size is set to 608 × 608 pixels. Before being fed into the network for feature extraction, the images undergo a series of preprocessing steps, including resizing, normalization, and padding, to meet the network’s input specifications. During training, YOLOv5 further enhances the input data through augmentation techniques such as Mosaic, which effectively enriches the feature diversity of the input images and thereby improves the model’s detection accuracy.

Baseline Network: The backbone of YOLOv5 primarily incorporates the Focus structure and the Cross-Stage Partial Network (CSPNet). The Focus module performs downsampling on the input image by aggregating spatial information from the width and height dimensions into the channel dimension, thereby enabling subsequent convolutional layers to extract more informative features. CSPNet, a lightweight network architecture, effectively enhances feature extraction by partitioning the feature map into two parts via a cross-stage connection strategy: one part is dedicated to feature transformation, while the other is preserved and later fused. This design improves the network’s accuracy and computational efficiency by balancing gradient flow and feature reuse.

Neck: The neck module of YOLOv5 comprises multiple upsampling and feature concatenation layers. These layers fuse feature maps extracted from the backbone (CSPNet) with multi-scale feature representations generated through a Feature Pyramid Network (FPN), thereby enhancing the detection of objects at varying scales. Notably, the upsampling layers within the neck adopt YOLOv5’s custom Cross Stage Partial (CSP) structure, which reduces memory consumption and improves computational efficiency. In addition, YOLOv5 integrates a Spatial Pyramid Pooling (SPP) module within the neck. The SPP structure captures spatial features at multiple receptive fields, generating multi-scale feature maps that support classification and regression tasks within the detection head. This design contributes to both improved detection accuracy and faster inference.

Detection Algorithm: Output Prediction Module. The detection component of the algorithm is responsible for generating the final prediction results. YOLOv5 employs a unique architecture incorporating Cross Stage Partial (CSP) connections and a Spatial Attention Module (SAM) to enhance its ability to detect objects of varying sizes and positions. Specifically, the detection mechanism integrates a strategy inspired by Fully Convolutional One-Stage Object Detection (FCOS), enabling effective object localization directly on the feature maps. Additionally, the Generalized Intersection over Union (GIoU) loss function is utilized to measure the overlap between the predicted bounding boxes and the ground truth, optimizing the loss and thereby improving detection accuracy.

#### 2.2.2. Attention Mechanism

##### SENet Attention Mechanism

Squeeze-and-Excitation Networks (SENet) introduce an attention mechanism that explicitly models interdependence and relative importance among feature maps. By adaptively recalibrating channel-wise feature responses, SENet enhances informative features while suppressing less useful ones, thereby improving the overall accuracy of the model.

**Figure 7:**
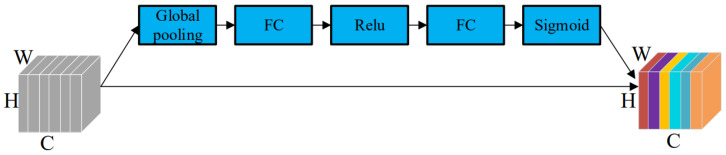
SE block attention mechanism module (Each color represents a channel in the input feature map, used to distinguish different feature channels.).

The core component of SENet is the Squeeze-and-Excitation (SE) block, which consists of a global average pooling layer, two fully connected (FC) layers, and a Sigmoid activation function. The structure of the SE block is illustrated in Figure 7.

Assuming the input feature map has dimensions H × W × C, where H, W, and C represent the height, width, and number of channels, respectively. The Squeeze-and-Excitation (SE) block begins by applying a global average pooling operation across the spatial dimensions (H × W), compressing each feature map into a single scalar. This results in a 1 × 1 × C channel descriptor vector, which captures the global spatial information of each channel and focuses the model’s attention on the channel-wise relationships. Next, this channel descriptor is passed through two fully connected (FC) layers, which form a bottleneck structure to model the inter-channel dependencies and relative importance. This process can be expressed as a parametric function $f\left(x\right)$, where x is the pooled feature vector and $f\left(x\right)=W\_2\delta (W\_1x)$. Here, W_1 and W_2 are the weight matrices of the two FC layers, and δ denotes a non-linear activation function (commonly ReLU). The output of this transformation is then passed through a Sigmoid activation function to constrain the values to the range [0, 1], producing a channel attention vector of size 1 × 1 × C. This vector represents the importance of each channel. Finally, the original feature map is recalibrated by performing channel-wise multiplication between the input feature map and the attention vector. This operation scales each channel by its learned importance weight, enabling the network to focus more on informative features while suppressing irrelevant or noisy channels. The SE block thus introduces an effective channel attention mechanism with negligible computational overhead, enhancing the model’s representational capacity and overall accuracy.

##### CBAM Attention Mechanism

**Figure 8:**
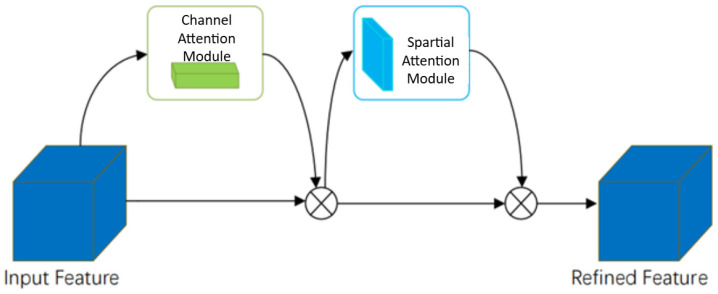
CBAM attention mechanism module.

The Convolutional Block Attention Module (CBAM) is an attention mechanism that combines both channel and spatial attention modules to enhance feature representation and improve image recognition accuracy, as illustrated in Figure 8. The CBAM module consists of two sequential submodules: the Channel Attention Module (CAM) and the Spatial Attention Module (SAM).

**Figure 9:**
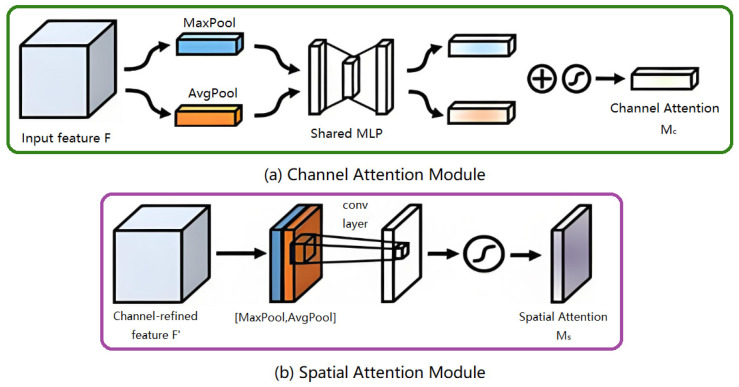
Channel attention and Spatial attention modules.

As shown in Figure 9a, the Channel Attention Module (CAM) is designed to enhance the representational power of feature channels. It achieves this by compressing the input feature map along the spatial dimension to obtain a channel-wise descriptor vector. This vector is then processed through two fully connected (FC) layers and an activation function to generate an attention vector, which is used to reweight each channel’s feature representation, allowing for the model to focus more on informative channel features. Specifically, average pooling and max pooling operations are applied independently along the spatial dimensions of the input feature map, producing two distinct descriptors. These are then merged and passed through two FC layers, followed by a sigmoid activation function to generate the channel attention vector. This vector is subsequently multiplied by the input feature map to adaptively adjust the importance of each channel.

As illustrated in Figure 9b, the Spatial Attention Module (SAM) enhances the spatial representational capability of feature maps by applying attention weights to each spatial location. This is achieved by compressing the input feature map along the channel dimension, generating two 2D spatial maps: one from average pooling and the other from max pooling operations. These two maps are then concatenated and passed through a convolutional layer, followed by a sigmoid activation function to produce a spatial attention map. The resulting spatial attention map is then element-wise multiplied with the original feature map, allowing for the network to adaptively emphasize informative regions while suppressing less relevant spatial areas.

The final output of the CBAM module is obtained by sequentially applying the channel attention module, followed by the spatial attention module, and combining their outputs. Since CBAM integrates both channel and spatial attention mechanisms, it demonstrates superior performance compared to models like SENet, which rely solely on channel attention. This dual attention strategy allows for CBAM to more effectively capture salient features across both dimensions.

##### Coordinate Attention (CA) Mechanism

**Figure 10:**
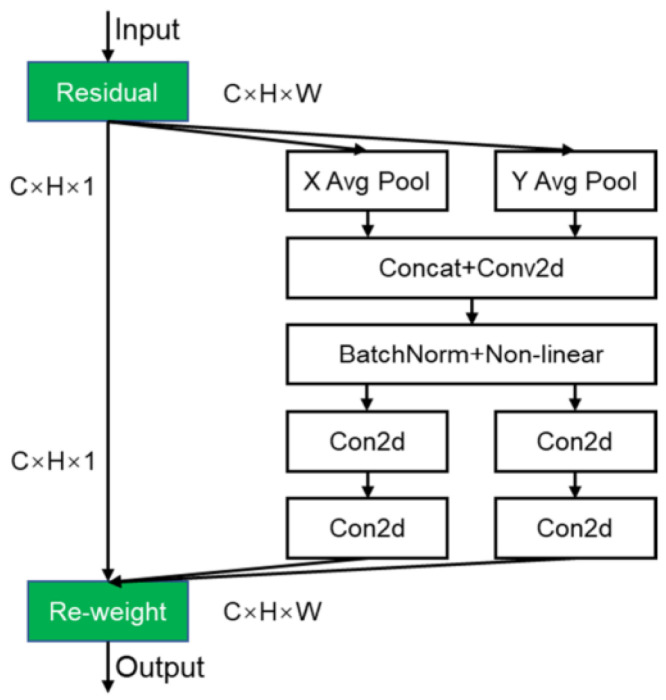
CA mechanism module.

Coordinate Attention (CA) is a spatial attention mechanism designed to enhance the representational capacity of feature maps within convolutional neural networks, as illustrated in Figure 10. Unlike SENet (Squeeze-and-Excitation) and CBAM (Convolutional Block Attention Module), which primarily focus on channel and spatial attention, respectively, CA specifically captures spatial positional information embedded in the input feature maps to improve the model’s ability to localize and recognize critical object regions.

The CA module consists of two components: horizontal attention and vertical attention. In the horizontal attention branch, the module applies one-dimensional global max pooling and average pooling operations along the horizontal axis to compute two feature vectors representing the maximum and average values of the feature map across the width dimension. These two vectors are then concatenated and passed through a fully connected layer, followed by a non-linear activation function such as ReLU, to produce a refined two-dimensional feature map referred to as the horizontal attention map.

In the vertical attention branch, the CA module similarly performs one-dimensional global max pooling and average pooling, but along the vertical axis. The resulting two feature vectors are processed through a fully connected layer followed by a non-linear activation function, generating a refined two-dimensional feature map known as the vertical attention map.

Finally, the CA module combines the horizontal and vertical attention maps into a unified attention map. This combined map encodes crucial spatial positional information as well as channel relationships, enabling the model to focus selectively on the most relevant regions and features for the given task.

In summary, by explicitly capturing spatial positional information within the feature maps, the Coordinate Attention mechanism enhances convolutional neural networks’ capacity to accurately localize and identify key target areas, thereby improving overall model performance.

#### 2.2.3. Improved YOLOv5

**Figure 11:**
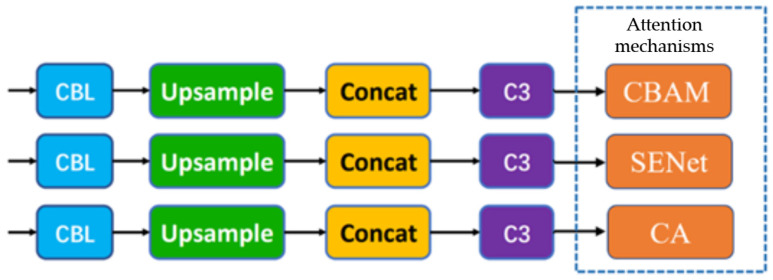
The attention mechanism adds location.

**Figure 12:**
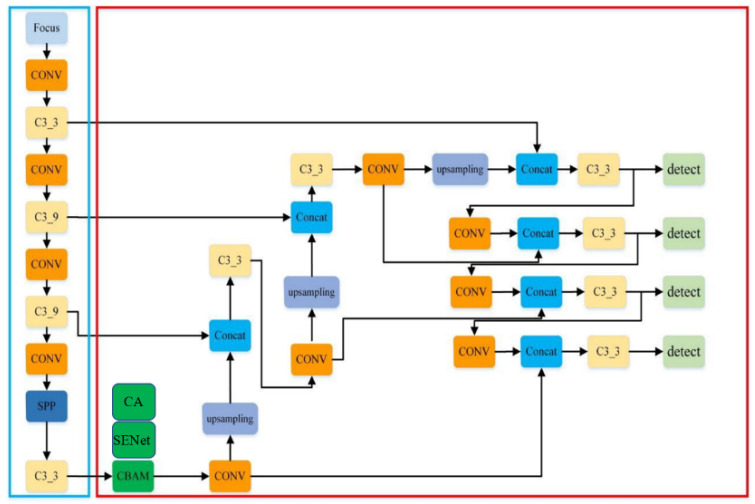
The improved YOLOv5 model structure(Orange represents convolution, light yellow represents output channel 3 or 9, green represents attention mechanism, light purple represents upsampling, blue represents concatenation, and light green represents detection).

Attention mechanisms are inspired by the human visual system, where the eye selectively focuses on salient regions within the field of view to allocate more cognitive resources for extracting important features while suppressing irrelevant information. Analogously, attention mechanisms in computer vision aim to enhance model performance by dynamically emphasizing crucial regions within an image, thereby improving the effectiveness of visual tasks. Due to their ability to provide fast and efficient feature representations, attention mechanisms have been widely adopted in the field of computer vision. To enable YOLOv5 to efficiently and effectively extract features from input images, this study integrates three distinct attention mechanisms—CBAM, SENet, and Coordinate Attention (CA)—into the neck module responsible for feature fusion. Comparative experiments were conducted using the baseline YOLOv5 network to evaluate and analyze the impact of these attention modules on model performance. The schematic diagram illustrating the placement of attention modules is shown in Figure 11, while the architecture of the improved YOLOv5 network incorporating these modules is presented in Figure 12.

## 3. Results

### 3.1. Experimental Setup

The experiments were conducted on a computer equipped with an AMD Ryzen 9 5950X processor and 192 GB of RAM. The models were implemented using the PyTorch (v2.1) deep learning framework and trained on an NVIDIA RTX 4090 GPU. The training process consisted of 300 epochs with a batch size of 8.

Using YOLOv5 as the baseline network model for *S. frugiperda* detection, the baseline model was trained with the same hyperparameters applied in subsequent experiments. The primary hyperparameters are summarized in Table 4.

### 3.2. Comparison of Benchmark Experiments

To comprehensively compare the performance of different deep learning networks in the task of *S. frugiperda* recognition, we systematically conducted a series of experiments and evaluations. Specifically, four representative object detection and instance segmentation frameworks—YOLOv7, YOLOv8, DETR, and Mask R-CNN—were trained and tested on the same *S. frugiperda* image dataset. These networks embody distinct architectural designs and are suited to different application scenarios: the YOLO series emphasizes detection speed and real-time performance, DETR leverages the global modeling capability of Transformers, while Mask R-CNN provides pixel-level segmentation alongside object recognition. For a fair comparison, we adopted mean Average Precision (mAP), number of parameters (Params), floating-point operations per second (FLOPs), and per-image inference time as evaluation metrics, thereby enabling a systematic analysis of the trade-offs among accuracy, computational complexity, and application efficiency.

As shown in Table 5 and Table 6, the experimental results demonstrate that different models exhibit distinct strengths in terms of accuracy, inference speed, and parameter scale. YOLOv7 and YOLOv8 offer advantages in detection speed, but tend to miss targets in scenarios with high insect density. DETR, which relies on the Transformer architecture, provides strong global modeling capability; however, its relatively slow inference speed limits practical applicability. Mask R-CNN is capable of pixel-level segmentation, yet it shows a high false detection rate in dense and overlapping insect scenarios and performs poorly in recognizing small targets, often misclassifying background elements or other insect species such as *S. frugiperda*. In contrast, YOLOv5 achieves a favorable balance between accuracy and real-time performance, maintaining a relatively high mean Average Precision (mAP) while simultaneously reducing parameter size and accelerating inference speed. Its lightweight and efficient design further enables stable deployment on resource-constrained embedded devices, thereby offering greater practical advantages. Consequently, this study ultimately adopts YOLOv5 as the baseline network for subsequent improvements and optimizations, providing a reliable experimental foundation for enhancing the recognition performance of *S. frugiperda*.

### 3.3. Experimental Results of the Attention-Enhanced YOLOv5 Algorithm

**Figure 13:**
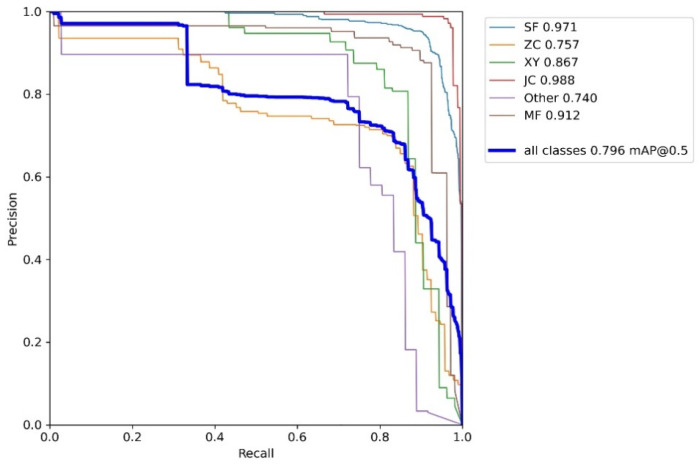
Add a PR graph for the CA mechanism.

**Figure 14:**
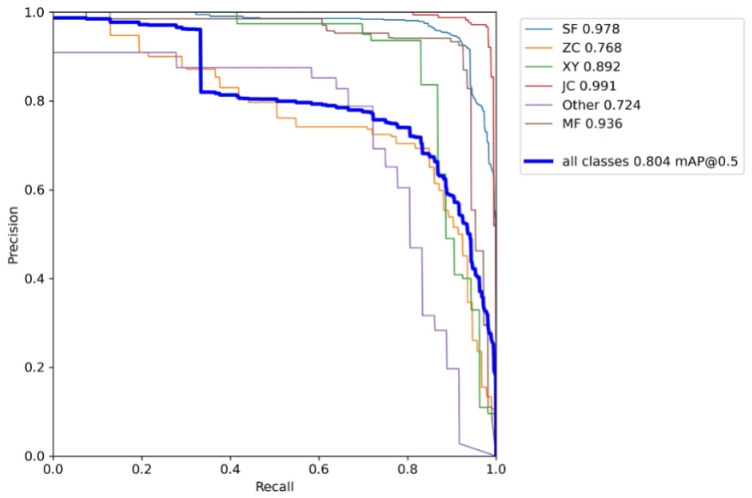
Add a PR graph for the CBAM attention mechanism.

**Figure 15:**
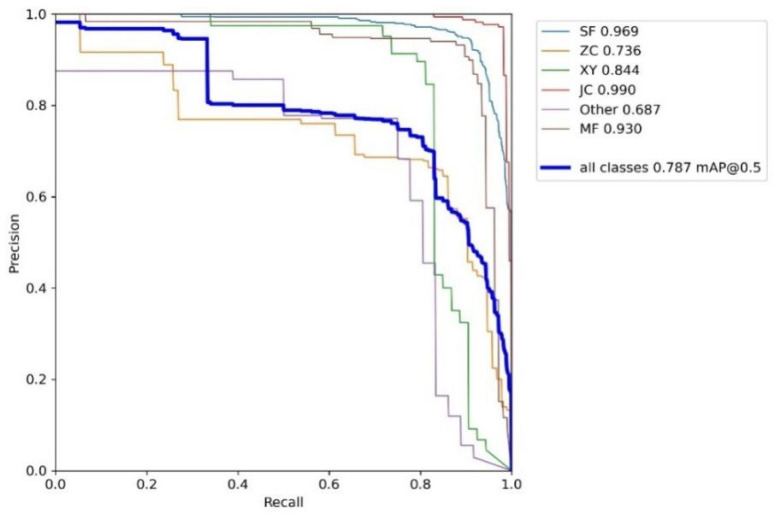
Add a PR graph for the SENet attention mechanism.

The *S. frugiperda* dataset was used to train the improved YOLOv5 models incorporating different attention mechanisms, including YOLOv5 integrated with Coordinate Attention (CA), Convolutional Block Attention Module (CBAM), and Squeeze-and-Excitation (SE) mechanisms. The hardware and software environment, as well as the experimental hyperparameter settings, were kept consistent with those of the baseline YOLOv5 model. The experimental results are summarized in Table 7, Table 8, Table 9 and Table 10, as well as Figure 13, Figure 14 and Figure 15.

The experimental results demonstrate that integrating the three different attention modules into the baseline YOLOv5 network led to improvements or maintenance in precision, recall, and mean average precision (mAP) across various insect categories in the trapped dataset. Specifically, with the Coordinate Attention (CA) module, the mean average precision improved for five insect categories—including *S. frugiperda*—except for the “other insects” category. Notably, *S. frugiperda*’s mAP increased by 0.1%, while *S. litura* exhibited the highest improvement with a 21% increase. With the Convolutional Block Attention Module (CBAM), all five major insect categories, excluding “other insects,” showed mAP gains, with the largest overall improvement among the three attention mechanisms. In particular, *S. frugiperda*’s mAP increased by 0.8%, and *S. litura* again demonstrated the most significant gain, improving by 23.5%. In contrast, the model augmented with the Squeeze-and-Excitation (SENet) attention module showed the smallest mAP improvement on the *S. frugiperda* dataset compared to the CA and CBAM modules. Moreover, the mAP for *S. frugiperda* slightly declined, although the decrease was marginal.

After incorporating different attention mechanisms (e.g., SENet, CBAM, and CA) into the model, the experimental results indicate that the overall number of parameters increased by only approximately 0.13 M. Such a marginal increment is negligible compared to the original YOLOv5s model and exerts minimal influence on computational complexity and storage requirements, thereby preserving the model’s lightweight characteristics. At the same time, the integration of attention mechanisms effectively enhances the model’s ability to represent and discriminate key features without compromising its lightweight nature, ultimately improving detection accuracy. This advantage is particularly critical in embedded systems, where computational power, memory, and energy consumption are severely constrained. The proposed improvement not only retains the efficiency and practicality of the model, but also ensures a balanced trade-off between real-time performance and accuracy, thereby achieving the fundamental objective of lightweight object detection.

### 3.4. A PyQt5-Based Visual Detection System for Adult S. frugiperda

**Figure 16:**
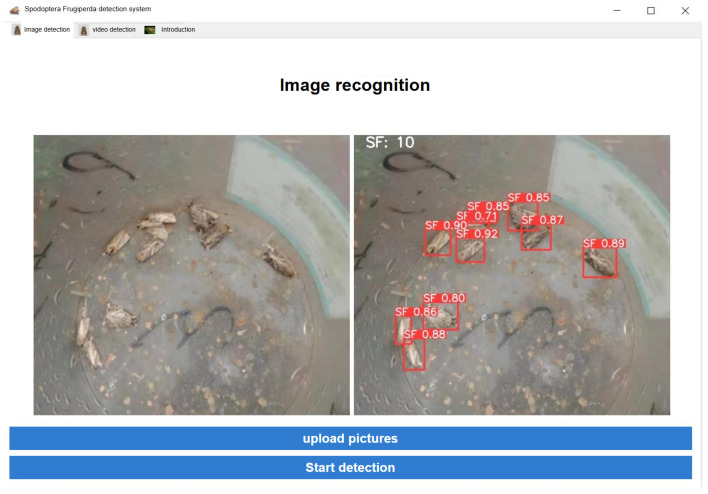
System image detection interface.

The visual interface for fall armyworm detection is shown in the figure below. As illustrated in Figure 16, the detection results demonstrate that the system accurately identifies and counts the target insects in the image. As shown, the system achieves accurate recognition and counting of target insects. The interface also includes an informational section about *S. frugiperda*, providing a brief introduction along with expandable content. By clicking on the prompt “Or you can learn more here,” users are redirected to the *S. frugiperda* page on Baidu Encyclopedia for further information. This system successfully integrates the PyQt5-based graphical user interface with the YOLOv5 detection model for *S. frugiperda*, enabling real-time detection and visualization of trapped fall armyworm images.

## 4. Discussion

This study focuses on the image-based recognition of trapped adult *S. frugiperda* and has yielded significant results while revealing areas for further improvement and reflection. The construction of a comprehensive dataset stands out as a pivotal achievement. By collecting images from multiple locations and diverse sources, we established a dataset encompassing various insect species. Previous studies have shown that geometric enhancement (such as random scaling and rotation) significantly improves small object detection [29], while color enhancement (such as HSV adjustment) can alleviate the interference of lighting changes [30]. Data augmentation techniques were further applied to balance the dataset, providing rich material for model training and laying a solid foundation for future research. In terms of model selection and enhancement, after comparing Mask R-CNN, YOLOv7, YOLOv8, DETR, and YOLOv5, we chose YOLOv5 as the baseline and introduced attention mechanisms for optimization. This approach effectively improved recognition accuracy. Specifically, the YOLOv5 model augmented with the CBAM attention mechanism achieved a mean Average Precision (mAP) of 97.8%, with a parameter count of only 7.33 M and an inference time of 0.0021 s per image(In a laboratory environment). The high efficiency and accuracy of the improved network enable stable operation on resource-constrained embedded devices. Additionally, the developed visual detection system integrates image recognition, counting, and information management functionalities, offering practical convenience for real-world applications. Nevertheless, certain limitations remain. From a data perspective, both the quantity and quality of images require further enhancement, as dataset size and quality directly influence model generalization. In real-world scenarios, the unpredictable movement and morphology of insects present challenges to accurate recognition, and research on video-based monitoring remains insufficient.

## 5. Future Work

This study systematically investigated the recognition of trapped *S. frugiperda* images using deep learning techniques, providing novel technical approaches and research ideas for the automatic identification and precise monitoring of this major agricultural pest. However, several limitations remain and should be addressed in future work. Firstly, the constructed *S. frugiperda* image dataset suffers from variable image quality due to differences in capturing device resolution, morphological diversity of trapped specimens, and uncertainties inherent in field image acquisition. These factors have constrained the overall dataset size and sample quantity, thereby limiting the training effectiveness and generalization capability of the deep learning models. Future research will focus on expanding the dataset scale, particularly increasing the proportion of high-quality images, and exploring diverse image enhancement and quality optimization techniques to improve dataset representativeness and robustness. Secondly, although YOLOv5, a widely adopted object detection algorithm, demonstrates excellent real-time detection performance, the dynamic characteristics of *S. frugiperda*—including flight behavior and resting postures—introduce considerable uncertainty in practical applications. Recognition based solely on static trapped images is insufficient to meet the demands of real-world pest monitoring. Considering the real-time requirements of pest surveillance, future studies will explore the integration of video acquisition and recognition frameworks. By leveraging temporal information from continuous frames, recognition stability can be enhanced. Provided sufficient video samples are available, a video-based recognition model for *S. frugiperda* will be developed to enable accurate monitoring in dynamic environments. Due to the current lack of accessible video data, this aspect was beyond the scope of the present study and constitutes a key direction for future investigation.

## 6. Conclusions

*S. frugiperda* is a major agricultural pest posing a serious threat to food security. Compared to traditional field manual monitoring, pest survey instruments, and trap-based methods, current pest monitoring techniques suffer from inaccurate identification and are time-consuming, failing to meet the urgent demand for efficient and precise pest surveillance in smart agriculture. To enhance the recognition capability and monitoring efficiency of *S. frugiperda*, this study established manual monitoring sites to collect field-trapped images of *S. frugiperda*, supplemented by laboratory and publicly available web samples, thereby constructing a dataset comprising six categories of trapped insects. To address sample imbalance, eleven image augmentation techniques were randomly combined to expand and balance the dataset, providing a stable foundation for model training. Based on this dataset, we developed and compared several detection models, including Mask R-CNN, YOLOv7, YOLOv8, DETR, and YOLOv5. The results demonstrate that YOLOv5 outperformed the other networks in terms of both detection accuracy and localization precision, while also exhibiting superior lightweight characteristics. Consequently, YOLOv5 can be regarded as a reliable baseline model. To further improve YOLOv5’s recognition accuracy and robustness, three attention mechanisms—SENet, CBAM, and Coordinate Attention (CA)—were integrated for model optimization. Experimental results demonstrate that the YOLOv5 model enhanced with CBAM attention achieved the best performance, reaching an average recognition precision of 97.8% on *S. frugiperda* trap images. This represents a significant improvement over the baseline, with notable gains in precision for four major insect categories, excluding the “Other” class. Finally, the CBAM-optimized YOLOv5 model was deployed within a PyQt5-based graphical user interface, completing the construction of an *S. frugiperda* detection system. The system integrates image recognition, result visualization, and pest information modules, enabling the automatic identification and counting of trapped *S. frugiperda* individuals. This provides an efficient and practical technical support tool for pest monitoring and intelligent pest management, with promising application prospects.

## Figures and Tables

**Table 1:** Characterization of the trap state of *S. frugiperda*.

The Morphological Characteristics of *S. frugiperda* in the Trapped State	Description of the Morphological Characteristics	Picture	Percentage of Images in the Dataset
Complete form	The shape features are complete, with the annular pattern and kidney-shaped pattern of the forewings clearly visible.	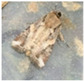	18%
Mild shedding of scales	Some shape features are missing, with prominent annular and kidney-shaped patterns on the forewings, while other features are faintly visible and the overall morphology is relatively complete.	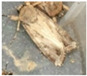	28%
Moderate shedding of scales	Some shape features are missing, the circular and kidney-shaped patterns on the forewings are not obvious, and other features are lost. The forewings are divided into alternating grayish white and grayish brown, and the head is starting to shed and turn red, with features no longer intact.	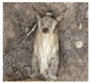	19%
The scales have completely fallen off	The morphological characteristics are completely lost, with the forewings being grayish white and the head being red.	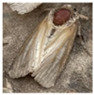	16%
Occlusion, overlap	The insects overlap and obscure each other.	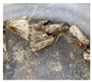	8%
flip	Due to the death of the *S. frugiperda*, the insect body flips over, revealing its abdomen.	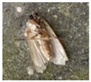	11%

**Table 2:** Sample data analysis table.

Category Number	Latin Name	Abbreviation	Number of Insects Before Equilibrium	Number of Insects After Equilibrium
1	*S.* *frugiperda*	SF	4673	4941
2	*Mythimna loreyi*	ZC	488	1201
3	*Spodoptera litura*	XY	421	896
4	*Apis*	MF	462	851
5	*Melolonthidae sensu stricto*	JC	720	1356
6	Other	Other	211	305
summation			6975	9550

**Table 3:** Statistics of the number of images collected in different environments.

Different Backgrounds and Lighting Conditions	Proportion of Images in the Dataset
Simple background and sufficient lighting	20%
The background is simple, and the lighting is insufficient	10%
Complex background and sufficient lighting	60%
Complex background and insufficient lighting	10%

**Table 4:** Training parameter setting of *S. frugiperda* model.

Parameter	Values
Images size	(608, 608, 3)
Epochs	300
Batch size	8
lr0	0.01
lrf	0.01
Weight decay coefficient	0.0005
Optimizer	Adam
Momentum	0.937

**Table 5:** Comparison of training results of different models.

Training Model	Category	mAP@0.5 (%)	Params (M)	FLOPs (G)	Times (in Seconds)
Mask R-CNN (ResNet50)	*S. frugiperda*	50.25%	44 M	85.5 G	0.0246 s
YOLOv7	*S. frugiperda*	75.54%	37 M	6.0 G	0.0072 s
YOLOv8	*S. frugiperda*	73.48%	68.2 M	8.7 G	0.0139 s
DETR (ResNet50)	*S. frugiperda*	68.33%	41 M	130 G	0.0333 s
YOLOv5	*S. frugiperda*	78.39%	7.2 M	2.6 G	0.0018 s

**Table 6:** Different model detection results.

Model	Actual Target Number	Number of Detections	Number of Missed Detections	Correctly Judge Numbers	Number of Incorrect Judgments	Detection Rate (%)	False Detection Rate (%)	Missed Inspection Rate (%)
Mask R-CNN	31	36	1	27	9	116	25	3
YOLOv7	31	32	3	27	5	103	15.6	9.6
YOLOv8	31	28	5	25	3	90.3	10.7	16
DETR	31	34	2	26	8	109	23.5	6.4
YOLOv5	31	32	0	28	4	103	12.5	0

**Table 7:** Experimental results of the *S. frugiperda* dataset adding the CA mechanism.

Class	Abbreviation	P	R	mAP@_0.5	mAP@_0.5:0.95
all	all	0.874	0.752	0.796	0.549
*S. frugiperda*	SF	0.949	0.904	0.971	0.71
*Mythimna loreyi*	ZC	0.726	0.742	0.757	0.552
*Spodoptera litura*	XY	0.873	0.792	0.867	0.589
*Melolonthidae sensu stricto*	JC	0.988	0.908	0.988	0.659
Other	Other	0.712	0.75	0.74	0.523
Apis	MF	0.92	0.832	0.912	0.541

**Table 8:** Experimental results of the *S. frugiperda* dataset with the CBAM attention mechanism.

Class	Abbreviation	P	R	mAP@_0.5	mAP@_0.5:0.95
all	all	0.868	0.78	0.804	0.55
*S. frugiperda*	SF	0.94	0.918	0.978	0.699
*Mythimna loreyi*	ZC	0.714	0.774	0.768	0.55
*Spodoptera litura*	XY	0.903	0.83	0.892	0.624
*Melolonthidae sensu stricto*	JC	0.982	0.932	0.991	0.652
Other	Other	0.609	0.778	0.724	0.488
Apis	MF	0.933	0.897	0.936	0.565

**Table 9:** Experimental results of the *S. frugiperda* dataset with the SENet attention mechanism.

Class	Abbreviation	P	R	mAP@_0.5	mAP@_0.5:0.95
all	all	0.863	0.771	0.787	0.546
*S. frugiperda*	SF	0.942	0.909	0.969	0.707
*Mythimna loreyi*	ZC	0.681	0.756	0.736	0.532
*Spodoptera litura*	XY	0.91	0.792	0.844	0.595
*Melolonthidae sensu stricto*	JC	0.982	0.928	0.99	0.653
Other	Other	0.683	0.778	0.687	0.497
Apis	MF	0.928	0.897	0.93	0.553

**Table 10:** Improved experimental comparison results based on YOLOv5.

Model	Class	P	R	mAP@_0.5	Params
YOLOv5	*S. frugiperda*	0.972	0.911	0.970	7.2 M
YOLOv5 + CA	*S. frugiperda*	0.949	0.904	0.971	7.3 M
YOLOv5 + CBAM	*S. frugiperda*	0.950	0.918	0.978	7.33 M
YOLOv5 + SENet	*S. frugiperda*	0.942	0.909	0.969	7.33 M

## Data Availability

The dataset used and/or analyzed during the current study is available from the corresponding author on reasonable request.
